# The Atlanta Medical and Surgical Journal

**Published:** 1855-08

**Authors:** 


### THE ATLANTA MEDICAL AND SURGICAL JOURNAL.

WE have received the prospectus of a journal bearing the above title, which is to be published at Atlanta, Ga., under the auspices of the Faculty of the Atlanta Medical College, at $3 per annum. It is to be edited by Joseph P. Logan, M. D., and W. F. Westmoreland, M. D., both Professors in the Atlanta school. From the names of the gentlemen who are to conduct it we may safely say that it will be devoted to the interests of legitimate medicine. Dr. Westmoreland is already known to the profession in the South as a laborious student and ready writer. We wish the gentlemen abundant success in their new and arduous enterprize.

